# Prevalence of underweight, overweight and obesity among children and adolescents in Germany. KiGGS Wave 2 results according to international reference systems

**DOI:** 10.17886/RKI-GBE-2018-087

**Published:** 2018-09-19

**Authors:** Anja Schienkiewitz, Stefan Damerow, Angelika Schaffrath Rosario

**Affiliations:** Robert Koch Institute, Berlin, Department of Epidemiology and Health Monitoring

**Keywords:** UNDERWEIGHT, OVERWEIGHT, OBESITY, INTERNATIONAL REFERENCE SYSTEMS, IOTF, WHO, HEALTH MONITORING

## Abstract

In Germany, the reference system according to Kromeyer-Hauschild is usually used to define underweight, overweight and obesity in children and adolescents. International classification systems to describe prevalence are the reference systems of the World Health Organization (WHO) and the International Obesity Task Force (IOTF). This article reports underweight, overweight and obesity prevalences among children and adolescents according to WHO and IOTF criteria using data from the second wave of the German Health Interview and Examination Survey for Children and Adolescents (KiGGS Wave 2, 2014-2017). According to the WHO reference system, the prevalence of underweight among 5- to 17-year-olds is 1.6%, the prevalence of overweight is 26.3% (including obesity) and the prevalence of obesity is 8.8%. According to IOTF, the prevalence of underweight among 3- to 17-year-olds is 10.0%. The prevalence of overweight (including obesity) is 19.3% and the prevalence of obesity is 4.7%. From a public health point of view, underweight as an indicator of malnutrition plays a rather minor role in Germany. The prevalence of overweight according to WHO is three quarters higher and one quarter higher according to IOTF than the national reference. When comparing the international reference systems, the WHO prevalence is one third higher than IOTF prevalence. According to national and international reference systems, no further increase in the prevalence of overweight and obesity is observed, but the prevalence remain at a high level.

## 1. Introduction

Children with overweight and obesity are more likely to have high blood pressure and disorders in lipid and glucose metabolism than children within the normal weight range [1]. A high body mass index (BMI) in childhood and adolescence is associated with a higher probability of type 2 diabetes, hypertension and cardiovascular disease in adulthood [2]. In addition, overweight and obesity among children and adolescents are associated with a significant reduction in quality of life [3] and with a higher risk of bullying [4]. Underweight reflects an insufficient nutritional status of children and is more prevalent in middle and low-income countries. It affects the growth of children, has negative implications on health at later stages in life and is associated with a higher mortality risk [5]. Worldwide, overweight and obesity as well as underweight are major public health problems in childhood and adolescence. Since the 1970s an increase in overweight and obesity prevalences among children and adolescents has been observed worldwide, a trend which as recently levelled off in high income countries [7-9] (as in Germany [6]). At the same time, the prevalence of underweight has decreased in most regions of the world. Overall, between 1975 and 2016, the increase in overweight and obesity preva lences was greater (girls +5 percentage points, boys +7 percentage points) than the decrease in the prevalence of underweight (girls -1 percentage point, boys -3 percentage points) [7].

**KiGGS Wave 2**

Second follow-up to the German Health Interview and Examination Survey for Children and Adolescents

**Data owner:** Robert Koch Institute

**Aim:** Providing reliable information on health status, health-related behaviour, living conditions, protective and risk factors, and health care among children, adolescents and young adults living in Germany, with the possibility of trend and longitudinal analyses

**Study design**: Combined cross-sectional and cohort study

**Cross-sectional study in KiGGS Wave 2**

**Age range:** 0-17 years

**Population:** Children and adolescents with permanent residence in Germany

**Sampling:** Samples from official residency registries - randomly selected children and adolescents from the 167 cities and municipalities covered by the KiGGS baseline study

**Sample size:** 15,023 participants

**KiGGS cohort study in KiGGS Wave 2**

**Age range:** 10-31 years

**Sampling:** Re-invitation of everyone who took part in the KiGGS baseline study and who was willing to participate in a follow-up

**Sample size:** 10,853 participants

**KiGGS survey waves**

► KiGGS baseline study (2003-2006), examination and interview survey
► KiGGS Wave 1 (2009-2012), interview survey
► KiGGS Wave 2 (2014-2017), examination and interview survey

More information is available at www.kiggs-studie.de/english

The definition of underweight, overweight and obesity is based on the BMI, which is an established index. It is calculated as body weight divided by the square of height (kg/m^2^) and is therefore fairly easy to measure. Since the ratio of body height and weight changes during childhood and adolescence, there is no uniform cut-off for all age groups for defining underweight, overweight and obesity.

The cut-off values for underweight, overweight and obesity in the age group up to 17 years are defined by percentiles as many other parameters in childhood and adolescence (see info box). An individual BMI value is considered relative to the BMI distribution in a defined group (reference population) taking into account age and gender. Thus girls and boys with particularly high (or low) values are evaluated in comparison to their peers. In Germany, categorisation is based on the Kromeyer-Hauschild et al. reference system [10, 11]. This defines a child with a BMI of 20 kg/m^2^ on its 7th birthday at the cut-off between overweight and obesity. However, a twelve-year-old child with a BMI of 20 kg/m^2^ would be of normal weight. For adults cut-offs are defined differently. The underlying factor here is the increased risk of diseases and higher mortality risks linked for example to a BMI greater than 30 kg/m^2^. Adults with a BMI of over 30 kg/m^2^ are classified as obese. For children and adolescents, the most common international reference systems to describe overweight and obesity, but also underweight, are the reference systems of the World Health Organization (WHO) [12, 13] as well as the International Obesity Task Force (IOTF) [14-16].

International reference systems not only allow comparisons of underweight, overweight and obesity prevalences between different countries, but also observations over time and thus a comparison of trends between countries. In this article, prevalences for underweight, overweight and obesity are calculated based on data from the second wave of the German Health Interview and Examination Survey for Children and Adolescents (KiGGS Wave 2) for the first time according to the international reference systems of the WHO and the IOTF. The results are discussed methodologically and evaluated to allow a presentation in the national and international context.

## 2. Methodology

### 2.1 Study design

KiGGS is part of the health monitoring system at the Robert Koch Institute and includes repeated cross-sectional surveys of children and adolescents aged 0 to 17, which are representative for Germany. The KiGGS baseline study was conducted as an examination and interview survey (2003-2006). KiGGS Wave 2 took place between 2014 and 2017 as a combined health examination and interview survey. The concept and design of KiGGS are described in detail elsewhere [17-20]. In brief, participants to be invited were selected randomly from the official population registries in 167 cities and municipalities representative for Germany and already used in the baseline study. A number of measures were taken to increase study participation and to improve the sample composition [17, 21]. The examination program of KiGGS Wave 2 started with children at the age of 3 years. 3,567 children and adolescents (1,801 girls, 1,766 boys) took part (participation rate 41.5%).

**Info box: Percentile curves**

Percentiles describe the distribution of continuous variables such as body height, weight or BMI within a reference population. This makes it possible to classify an individual value in the context of age and gender during childhood and adolescence. A given percentile reading from a growth curve indicates the percentage of children of the same age and gender that are below this value.

**Figure:**
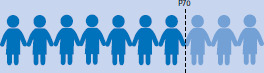

If the body weight of an 8-year-old girl is the 70th percentile (P70), 70% of 8-year-old girls have a lower body weight and 30% of girls of this age have a higher body weight.

### 2.2 Underweight, overweight and obesity according to different reference systems

In the examination part of KiGGS Wave 2, standardised measurements of body weight and height were obtained. BMI (kg/m^2^) was calculated from body weight and height. Prevalences of underweight, overweight and obesity were determined according to the Kromeyer-Hauschild reference system, as well as the WHO and IOTF international reference systems.

According to the German reference system by Kromeyer-Hauschild et al. [10, 11], children and adolescents are classified as underweight if their BMI value is below the 10th percentile (see info box). Underweight thereby includes severe underweight (below the 3rd percentile). A BMI value above the 90th percentile is defined as overweight and BMI values above the 97th percentile as obesity. Therefore, overweight is defined by including obesity. Under this statistical definition, 10% of children in the reference population are classified as underweight, 10% as overweight and 3% as obese. The WHO and IOTF reference systems use other reference populations and have determined different percentiles to define cut-offs (Table 1 and Table 2).

The WHO reference system consists of the WHO growth standard for younger children under five years of age [12] and the WHO reference for children and adolescents aged 5 to 19 years [13]. The growth standard is based on cross-sectional and longitudinal data collected in the Multi Growth Reference Study (MGRS). MGRS is a population-based study that was conducted between 1997 and 2003 in Brazil, Oman, Norway, Ghana, India and the US. Included in the study were children (n=8,440) who were breastfed for four months and lived in good socioeconomic conditions. For children under 5 years of age, the WHO growth standard does not use BMI in relation to age to define underweight, overweight and obesity, but rather body height and body weight in relation to age, as well as the body weight in relation to body height [12].

The WHO reference for children and adolescents aged 5 years and older is based on data from the US National Center for Health Statistics, which were collected from approximately 22,000 children, adolescents and young adults aged 1 to 24 years between 1963 and 1975. For these older children and adolescents, BMI provides the basis of the reference system. For 19-year-olds, the cut-offs for overweight (BMI ≥ 25.0 kg/m^2^) and obesity (BMI ≥ 30.0 kg/m^2^) are nearly the same as those for adults [13].

The WHO growth standard and the WHO reference are based on age and gender-specific percentile curves (see info box). The definition of cut-offs is expressed as standard deviations (SD) from the median. SD values can also be rendered as percentiles, although this presentation is rather unusual for WHO cut-offs. For example, a value of +2 SD corresponds to the 97.7th percentile. This means that 97.7% of the values for children of the same age and gender are below this value and 2.3% are above (Table 1).

For children under five years of age, WHO uses three different parameters to assess an excessively low body weight or body height (Table 1). The first parameter is weight-for-age, i.e. the distribution of body weight in relation to age. In this age group, WHO defines children as underweight whose weight is below the P2.3 percentile.

Secondly, WHO considers stunting, a state of chronic nutritional deficiency that impaired growth of body height. Stunting is assessed by the parameter height-for-age, i.e. body height in relation to age. Stunting is defined as a height below percentile P2.3 (whereby the child looks ‘normal’ with regard to body height and weight, but is too small for its biological age).

The third parameter is wasting and refers to the parameter weight-for-height, i.e. sets body weight in relation to body height. In contrast to stunting, wasting indicates an acute deficiency. It is defined as a body weight below percentile P2.3 compared to children of the same height. The definitions of overweight and obesity for under-5-year-olds also refer to body weight in relation to height (weight-for-height) and are defined as percentile P97.7 or percentile P99.9, respectively [12].

For children and adolescents from 5 to 19 years of age, WHO uses BMI in relation to age to define underweight, overweight and obesity. Percentile P2.3 is defined as underweight, P0.13 as severe thinness, P84.0 as overweight and P97.7 as obesity. Underweight includes all forms of thinness and severe thinness, overweight is defined including obesity.

The IOFT reference system (Table 2) is based on data from nationally representative cross-sectional surveys from six countries (Brazil, the UK, Hong Kong, the Netherlands, Singapore and the US) with more than 10,000 participants aged 0 to 25 [14, 15]. Individual BMI values were modelled for girls and boys aged 2 to 18 years and growth curves were established. The cut-offs for determining underweight, overweight and obesity were not chosen as prescribed SD values or percentiles, but ‘bounded’ to adult cut-offs. For example, to define obesity at the age of 18 years the percentile corresponds to the adult cut-off value of 30 kg/m^2^, this was rounded P99. For overweight this procedure yielded approximately the 90th percentile as cut-off – the percentile, which is also used in other reference systems to define overweight in childhood and adolescence. Overweight thereby includes all subjects with obesity and morbid obesity. Underweight includes all grades of thinness [16]. The IOTF reference system is defined for the 2 to 18 age group.

### 2.3 Statistical analysis

The analyses are based on data from 3,561 participants (1,799 girls and 1,762 boys) aged 3 to 17 years with valid measurements of body height and weight. Six participants were excluded due to missing values in body weight and/or height. The results were stratified by gender and different age groups or cohorts and are presented as prevalences with 95% confidence intervals (95% CI). A corresponding weighting factor was used in order to make representative statements taking into account the regional structure, age (in years), gender, federal state (official population as of 31 December 2015), German citizenship (as of 31 December 2014) as well as parent levels of education according to the Comparative Analysis of Social Mobility in Industrial Nations (CASMIN) [22] classification (Microcensus 2013 [23]).

All analyses were conducted with SAS 9.4 (SAS Institute, Cary, NC, US) using the KiGGS Wave 2 data (Version 09). Survey procedures for complex samples were used in all analyses to adequately account for the clustering of participants in sample points and to consider weighting in the calculation of confidence intervals and p-values [9].

A statistically significant difference between girls and boys or between age cohorts is assumed when the corresponding p-value is smaller than 0.05.

## 3. Results

### Prevalences according to the WHO reference system

In the examination part of KiGGS Wave 2 children aged 3 years and older were included. Therefore, data on height and weight for the age group under five years are only available for 3- and 4-year-old children. The prevalence of underweight according to the WHO reference system is 0.5% for this age group (Table 3). Wasting (low weight in relation to height, see above) affects 0.3% of children. The prevalence rate of stunting (low height in relation to age, see above) is estimated at 1.7%. No statistically significant differences were found between girls and boys. The prevalence of overweight (including obesity) is 3.2%. Based on this definition, significantly more girls (5.9%) are overweight than boys (0.7%). According to the WHO reference system, 0.1% of children aged 3 to 4 years are obese. As this age group comprises only two cohorts in KiGGS Wave 2, the confidence intervals for these prevalences are very broad. This indicates a great statistical uncertainty in the results.

According to the WHO reference system for the age group 5 to 17 years, a prevalence of 1.6% is determined for underweight based on KiGGS Wave 2 data (Table 4). A small proportion of adolescents is affected by severe thinness (0.3%). There are neither differences between girls and boys, nor between the age groups. 26.3% of children and adolescents are overweight (or obese) and 8.8% are obese. In the age group 5 to 17 years, significantly more boys than girls are affected by overweight or obesity. The highest prevalences of overweight and obesity are found in the age group 11 to 13 years (girls 29.3% and boys 35.6%), while prevalences of obesity did not differ statistically significantly between the age groups.

### Prevalences according to the IOTF reference system

According to IOTF, the prevalence of underweight among girls and boys aged 3 to 17 years is 10% (Table 5). In this age group, severe thinness affects 1.5% of children and adolescents. The prevalence of overweight (including obesity) is 19.3%. Obesity prevalence is 4.7%. Morbid obesity even affects 1.0% of girls and boys. There are no statistically significant gender differences.

Among girls and boys, age has no statistically significant impact on the prevalence of underweight. The prevalence of overweight, obesity and morbid obesity increases among girls and boys with increasing age. However, this increase is only statistically significant among boys.

According to the IOTF definition, the prevalence of overweight is highest for the 11- to 13-year-old girls (23.9%) and boys (28.4%). The highest prevalence of obesity is present among 14- to 17-year-olds and is 5.7% among girls and 5.8% among boys. The highest prevalence of morbid obesity is also found in the age group 14 to 17 years.

## 4. Discussion

The aim of this article is to describe prevalences of underweight, overweight and obesity according to the inter national reference systems of WHO and the IOTF reference system and to evaluate them in the international context. In Germany, this is made possible by nationwide data on body height and weight of children and adolescents from KiGGS Wave 2 (2014-2017).

Based on the national reference system commonly used in Germany by Kromeyer-Hauschild et al. [10, 11], the current results of KiGGS Wave 2 indicate that 15% of girls and boys aged 3 to 17 years are affected by overweight, the prevalence of obesity is 6% [6]. According to the WHO reference system for the age group 5 to 17 years, 26% of children and adolescents are overweight and 9% obese. Using the IOTF reference system 19% of 3- to-17-year-olds are overweight and 5% obese.

The prevalence of overweight is thus three quarters higher than the WHO reference and one quarter higher according to IOTF compared to the national reference system. If the international reference systems are compared, the prevalence according to WHO is one third higher than according to IOTF. As for obesity, the prevalence is lower under IOTF and higher under WHO compared to the national reference system. Higher WHO prevalence estimates compared to IOTF were also found in other countries that conducted representative surveys [24, 25] and non-representative regional cross-sectional studies [26, 27]. The reason for this is that cut-offs according to WHO from five years onward are consistently lower than IOTF cut-offs.

However, the higher prevalences of overweight and obesity according to the WHO reference system apply only for older children and adolescents. Among children under five years, the WHO cut-offs for overweight are higher compared to the IOTF reference system, and thus lead to lower prevalences. This is due to the application of the WHO growth standard for children under five years and the WHO reference for children and adolescents aged 5 to 19 years. For children under five years, the WHO standard is derived from a population presenting optimal growth. This approach is based on the concept that under ideal conditions the average growth of a child is the same all over the world. It thus differs significantly from the underlying study population of WHO reference values for older children and adolescents, which were generated exclusively on regional data from the USA without taking into account other study populations and the general health status.

Furthermore, there are differences in the definition of overweight and obesity and the choice of cut-offs (weight-for-height P97.7 or P99.9) for girls and boys aged under five years compared to older children and adolescents (BMI-for-age P84.0 or P97.7). Thus, not only does the WHO reference indicate a sharp increase in prevalences at the transition from four to five years of age, but there are also significant differences between the WHO standard and the IOTF references in the age group of children under five years of age. One reason for this is that that WHO does not want to categorise young children too hastily as being either overweight or obese.

Although children and young people from different population groups have similar growth patterns, the cut-offs are set on varying percentiles. The classification of children in comparison to their peers therefore differs between the reference systems, and these differences influence prevalence estimates.

Despite various definitions of underweight, overweight and obesity according to IOTF and WHO, absolute differences between countries regarding the prevalences of overweight and obesity presumably exist. Within Europe, for example, obesity prevalences in childhood and adolescence vary between 12% and 40% [28]. The WHO estimates published for overweight and obesity in north-western Europe are however more or less in line with the KiGGS Wave 2 results according to WHO [7]. A direct comparison of KiGGS Wave 2 results with prevalences of other countries is methodologically difficult. A publication that would allow a classification of KiGGS data in a European context is in progress [29]. Comparisons of prevalence across national borders remain complicated because there is no internationally uniform and globally valid reference system. However, it is at least possible to compare prevalences in a reference system over time.

### Limitations

Using prevalences according to WHO for children under five years of age it is necessary to consider that KiGGS Wave 2 only provides data for children aged over three years and that prevalences for children under five years are therefore only calculated with the data of children aged three and four years. For children and adolescents aged 5 to 17 measurements are available.

Moreover, it cannot be ruled out that prevalence estimates for obesity are biased, as adolescents and young adults in extreme weight categories appear to be less willing to participate in surveys [30, 31].

### Public health relevance

According to the WHO, underweight affects less than 2% of children and adolescents in Germany. The WHO’s Nutrition Landscape Information System defines cut-off values for the indicators underweight, wasting and stunting which have major public health significance. An underweight prevalence less than 10% and prevalence of stunting of less than 20% are considered low. A prevalence of wasting of less than 5% is regarded acceptable. Wasting and stunting are among the WHO 100 core health indicators and are regularly collected and reported to the WHO. They are continuously monitored within the context of the Global Nutrition Targets to improve the nutrition of mothers, infants and young children. They provide concise information on the country’s health situation and contribute to monitoring and evaluating the country’s priorities to improve health care services [32]. In Germany, in comparison to overweight and obesity, underweight plays a minor role as an indicator of malnutrition from a public health perspective and is more frequently considered within the context of eating disorders. Overweight and in particular obesity are associated with long-term unfavourable health outcomes. With its Global Action Plan for the Prevention and Control of Non-Communicable Diseases (NCD), WHO declared the goal to “halt the rise in obesity” by 2025 [33]. According to the national reference system, Germany has achieved this target [6]. This also applies according to the international reference systems. If the calculation of prevalences in the KiGGS baseline study is adjusted to the age range, age structure and weighting procedure of KiGGS Wave 2, the prevalence of overweight according to IOTF was 19.8% and for obesity 5.6% in the KiGGS baseline study (2003-2006) and thus even slightly higher than currently observed in KiGGS Wave 2 [34]. The application of WHO references for the 5- to 17-year-olds provides the same result. The prevalence of overweight according to WHO in the KiGGS baseline study was, adapted to the current age and weighting structure, 26.9% and the prevalence of obesity was 9.3%.

This indicates that the use of different reference systems can lead to consistent results over time trends, even if the absolute prevalences differ between reference systems. For the comparison of trends between countries, it therefore makes sense (but is not absolutely necessary) to use the same reference systems. However, to compare prevalences and absolute figures between countries, it is essential to apply international classification systems [14]. Moreover, it also makes sense to use international reference systems if no national system is available.

In Germany, however, the national classification system is the better option to indicate clinical needs, for example regarding the therapeutic choices for those diagnosed as under- or overweight. A systematic review has convincingly demonstrated that using national reference data to categorise a high BMI and/or the corresponding percentile is more suitable for diagnosing obesity than the assessment applying international reference systems [35]. Therefore, international classification systems are less suitable for clinical use, as the underlying study populations are very heterogeneous.

### Conclusion

Underweight, overweight and obesity can already lead to health problems in early childhood, with consequences which continue to progress into adulthood. The WHO target to halt the rise in obesity among children and adolescents has thus been achieved in Germany, but prevalences remain at a high level. In this context, it is necessary to describe the development of these indicators, which are important from a public health perspective, and to contextualise them internationally, against the background of methodological difficulties.

## Key statements

- According to the WHO reference system, the prevalence of underweight among 5- to 17-year-olds is 1.6%, the prevalence of overweight is 26.3% (including obesity) and the prevalence of obesity is 8.8%.
- According to IOTF, the prevalence of underweight among 3- to 17-year-olds is 10.0%, the prevalence of overweight (including obesity) is 19.3% and the prevalence of obesity is 4.7%.
- From a public health point of view, underweight as an indicator of malnutrition plays a rather minor role in Germany.
- Using national as international reference systems, no further increase in overweight and obesity prevalence over time is observed – nevertheless these prevalences remain at a high level.

## Figures and Tables

**Table 1.** Definition of underweight, overweight and obesity according to the WHO reference system Source: WHO Multicentre Growth Reference Study Group (2006) [12], de Onis et al. (2007) [13]

WHO category	Parameter	Cut-off as SD	Cut-off as percentile
**Children under five years of age**			
Underweight	weight-for-age	< -2 SD	P2.3
Wasting	weight-for-height	< -2 SD	P2.3
Stunting	height-for-age	< -2 SD	P2.3
Overweight	weight-for-height	> +2 SD	P97.7
Obesity	weight-for-height	> +3 SD	P99.9
**Children and adolescents 5 to 19 years of age**			
Severe thinness	BMI-for-age	< -3 SD	P0.13
Thinness	BMI-for-age	< -2 SD	P2.3
Overweight	BMI-for-age	> +1 SD	P84.0
Obesity	BMI-for-age	> +2 SD	P97.7

WHO = World Health Organization, SD = standard deviation, P = percentile, BMI = body mass index

**Table 2.** Definition of underweight, overweight and obesity according to the IOTF reference system Source: Cole & Lobstein (2012) [16]

IOTF category	BMI cut-off age 18 years and older	Equivalent percentile for girls/boys
Thinness Grade 3 (Severe thinness)	< 16.0 kg/m^2^	P0.7/P0.5
Thinness Grade 2 (Wasting)	< 17.0 kg/m^2^	P3.7/P3.0
Thinness Grade 1	< 18.5 kg/m^2^	P16.5/P15.5
Overweight	≥ 25.0 kg/m^2^	P89.3/P90.5
Obesity	≥ 30.0 kg/m^2^	P98.6/98.9
Morbid obesity	≥ 35.0 kg/m^2^	P99.8 for both

IOTF = International Obesity Task Force, BMI = Body Mass Index, P = percentile

**Table 3.** Underweight, overweight and obesity according to the WHO reference system for children under five* years according to gender (n=215 girls, n=221 boys) Source: KiGGS Wave 2 (2014-2017)

	Underweight weight-for-age < -2 SD		Wasting weight-for-height < -2 SD		Stunting height-for-age < -2 SD		Übergewicht weight-for-height > +2 SD		Adipositas weight-for-height > +3 SD	
	%	(95% Cl)	%	(95% Cl)	%	(95% Cl)	%	(95% Cl)	%	(95% Cl)
**Total**	**0.5**	**(0.1-2.4)**	**0.3**	**(0.0-2.4)**	**1.7**	**(0.7-3.7)**	**3.2**	**(1.7-6.1)**	**0.1**	**(0.02-1.0)**
Girls	0.2	(0.0-1.7)	XX	XX	2.2	(0.8-5.9)	5.9	(2.9-11.7)	XX	XX
Boys	0.8	(0.1-5.2)	0.7	(0.0-4.6)	1.2	(0.3-4.8)	0.7	(0.2-3.0)	0.3	(0.0-2.1)
p-value**	n. s.		---		n. s.		0.0024		---	

WHO = World Health Organization, CI = confidence interval, SD = standard deviation, n. s. = not significant

XX = no figures, --- = no p-value calculated

^*^ KiGGS Wave 2 data available only for children aged three and four years

^**^ p-value for gender differences

**Table 4.** Underweight, overweight and obesity according to the WHO reference system for children and adolescents aged 5 to 17 years according to gender and age Source: KiGGS Wave 2 (2014-2017)

	n	Severe thinness BMI-for-age < -3 SD		Thinness BMI-for-age < -2 SD		Overweight BMI-for-age > +1 SD		Obesity BMI-for-age > +2 SD	
		%	(95% Cl)	%	(95% Cl)	%	(95% Cl)	%	(95% Cl)
**Total**	**3,125**	**0.3**	**(0.1-0.6)**	**1.6**	**(1.1-2.2)**	**26.3**	**(24.2-28.5)**	**8.8**	**(7.5-10.3)**
5-10 Years	1,355	0.1	(0.0-0.3)	1.1	(0.6-1.8)	24.9	(22.0-28.1)	9.4	(7.4-11.8)
11-13 Years	815	0.7	(0.2-2.2)	2.2	(1.2-3.8)	32.5	(28.0-37.5)	9.3	(6.7-12.6)
14-17 Years	955	0.3	(0.1-1.1)	1.9	(1.1-3.5)	23.9	(20.8-27.3)	7.8	(5.8-10.4)
p-value*		n. s.		n. s.		0.0033		n. s.	
**Girls**	**1,584**	**0.2**	**(0.0-1.1)**	**1.1**	**(0.6-1.9)**	**23.6**	**(20.8-26.5)**	**6.7**	**(5.2-8.6)**
5-10 Years	648	XX	XX	0.6	(0.2-1.5)	21.3	(17.5-25.7)	7.4	(5.0-10.9)
11-13 Years	410	0.9	(0.2-4.7)	2.6	(1.1-6.1)	29.3	(23.6-35.6)	6.3	(3.6-10.9)
14-17 Years	526	XX	XX	0.7	(0.3-1.6)	22.7	(18.4-27.5)	5.9	(3.7-9.3)
p-value*		---		0.0098		n. s.		n. s.	
**Boys**	**1,541**	**0.3**	**(0.1-0.8)**	**2.1**	**(1.4-3.1)**	**28.9**	**(25.6-32.4)**	**10.8**	**(8.8-13.3)**
5-10 Years	707	0.1	(0.0-0.6)	1.5	(0.8-2.8)	28.3	(23.8-33.2)	11.2	(8.2-15.1)
11-13 Years	405	0.4	(0.1-1.9)	1.7	(0.9-3.4)	35.6	(29.1-42.8)	12.0	(8.0-17.7)
14-17 Years	429	0.5	(0.1-2.2)	3.0	(1.5-5.9)	25.0	(20.4-30.3)	9.5	(6.5-13.7)
p-value*		n. s.		n. s.		0.0308		n. s.	
p-value**		n. s.		n. s.		0.0216		0.0045	

WHO = World Health Organization, BMI = body mass index, CI = confidence interval, SD = standard deviation, n. s. = not significant

XX = no entry, --- = no p-value calculated

^*^ p for age group differences

^**^ p-value for gender differences

**Table 5.** Underweight, overweight and obesity according to the IOTF reference system for children and adolescents aged 3 to 17 years according to gender and age Source: KiGGS Wave 2 (2014-2017)

	n	Severe thinness (Grade 2/3) BMI < 17,0 kg/m^2^		Thinness (Grade 1) BMI < 18,5 kg/m^2^		Overweight BMI > 25,0 kg/m^2^		Obesity BMI > 30,0 kg/m^2^		Morbid obesity BMI > 35,0 kg/m^2^	
		%	(95% Cl)	%	(95% Cl)	%	(95% Cl)	%	(95% Cl)	%	(95% Cl)
**Total**	**3,561**	**1.5**	**(1.1-2.0)**	**10.0**	**(8.8-11.4)**	**19.3**	**(17.4-21.4)**	**4.7**	**(3.8-5.7)**	**1.0**	**(0.7-1.6)**
3-6 Years	880	1.9	(1.2-3.2)	12.4	(9.9-15.3)	10.8	(8.3-14.0)	1.8	(1.0-3.4)	0.3	(0.1-0.8)
7-10 Years	911	0.4	(0.2-0.9)	10.4	(8.1-13.3)	19.6	(16.3-23.4)	5.3	(3.5-7.8)	0.5	(0.1-2.3)
11-13 Years	815	2.0	(1.0-3.6)	8.1	(6.1-10.8)	26.2	(22.0-31.0)	5.9	(3.8-9.1)	1.1	(0.5-2.7)
14-17 Years	955	1.7	(0.9-3.2)	8.8	(6.5-11.7)	21.7	(18.7-25.1)	5.8	(4.1-8.1)	2.0	(1.1-3.7)
p-value*		0.0460		n. s.		<0.0001		0.0108		0.0200	
**Girls**	**1,799**	**1.5**	**(1.0-2.4)**	**8.9**	**(7.3-10.9)**	**19.2**	**(16.8-21.9)**	**4.6**	**(3.5-6.0)**	**1.1**	**(0.6-2.0)**
3-6 Years	426	1.6	(0.8-3.1)	9.1	(6.7-12.4)	14.4	(10.2-20.1)	3.0	(1.4-6.1)	0.5	(0.2-1.6)
7-10 Years	437	0.6	(0.2-1.8)	10.4	(7.0-15.2)	18.8	(14.3-24.2)	4.5	(2.7-7.4)	XX	XX
11-13 Years	410	2.6	(1.1-6.1)	8.4	(5.6-12.3)	23.9	(18.6-30.2)	5.1	(2.6-9.8)	0.8	(0.1-4.7)
14-17 Years	526	1.6	(0.6-4.0)	7.8	(5.1-11.9)	20.5	(16.5-25.3)	5.7	(3.6-9.1)	2.7	(1.2-6.0)
p-value*		n. s.		n. s.		n. s.		n. s.		---	
**Boys**	**1,762**	**1.4**	**(0.9-2.1)**	**11.0**	**(9.2-13.0)**	**19.4**	**(16.6-22.5)**	**4.8**	**(3.5-6.4)**	**1.0**	**(0.5-1.8)**
3-6 Years	454	2.3	(1.2-4.3)	15.4	(11.3-20.7)	7.4	(4.8-11.2)	0.7	(0.2-2.4)	XX	XX
7-10 Years	474	0.2	(0.0-0.7)	10.5	(7.3-14.9)	20.4	(15.5-26.4)	6.0	(3.4-10.2)	1.0	(0.2-4.5)
11-13 Years	405	1.3	(0.6-3.0)	7.9	(5.4-11.4)	28.4	(22.2-35.6)	6.7	(3.7-11.9)	1.5	(0.6-3.8)
14-17 Years	429	1.8	(0.8-4.1)	9.7	(6.5-14.2)	22.8	(18.2-28.1)	5.8	(3.6-9.3)	1.4	(0.6-3.5)
p-value*		0.0404		n. s.		<0.0001		0.0088		---	

IOTF = International Obesity Task Force, BMI = body mass index, CI = confidence interval, n. s. = not significant XX = no entry, --- = no p-value calculated

^*^ p-value for age group differences
